# Supplementary material for: Mapping the evolution of fertility support policies in China: A content and instrumental analysis
Source: PLoS One. 2025 Oct 9;20(10):e0332137. doi: 10.1371/journal.pone.0332137 (PMC12510515; doi:10.1371/journal.pone.0332137)
Supplement: S1 Appendix — (ZIP) [file pone.0332137.s001.zip › S1 Appendix. 226 original policy documents/145-中国妇女发展纲要(FBM-CLI-2-12670).doc]

中国妇女发展纲要

**发布部门：** [国务院](https://www.pkulaw.com/law/chl?Aggs.IssueDepartment=5)

**发布日期：**1995.07.27

**实施日期：**1995.07.27

**时效性：** [现行有效](https://www.pkulaw.com/law/chl?Aggs.TimelinessDic=01)

**效力级别：** [国务院规范性文件](https://www.pkulaw.com/law/chl?Aggs.EffectivenessDic=XC0203)

**法规类别：** [老少妇幼残保护](https://www.pkulaw.com/law/chl?Aggs.Category=01604)

国务院关于印发中国妇女发展纲要（1995~2000年）的通知
（国发[1995]23号）

各省、自治区、直辖市人民政府，国务院各部委、各直属机构：
　　为进一步促进中国妇女的进步和发展，国务院妇女儿童工作委员会在调查研究、广泛征求意见的基础上，编制了《中国妇女发展纲要（1995~2000年）》（以下简称《纲要》）。国务院同意这一《纲要》，现印发给你们，请认真贯彻执行。
　　《纲要》明确了今后6年我国妇女发展的任务和主要目标，提出了相应措施。各省、自治区、直辖市人民政府要根据《纲要》的要求，结合实际情况制定本地区的妇女发展规划。各级人民政府和各有关部门，要采取切实措施，努力实现《纲要》提出的各项任务。

国务院
一九九五年七月二十七日

中国妇女发展纲要
（１９９５－２０００年）

序言

　　一、妇女是创造人类文明和推动社会发展的一支伟大力量。妇女的发展水平，是社会发展的重要指标，也是衡量社会进步程度的尺度。促进我国妇女的进步和发展，是各级政府、各有关部门、各社会团体和全国人民的共同任务。为了进一步促进我国妇女的发展，特制定《中国妇女发展纲要（１９９５－２０００年）》（以下简称《纲要》）。

　　二、新中国成立４６年来，我国妇女事业取得了举世瞩目的成就。目前，已经基本形成了以《[中华人民共和国宪法](https://www.pkulaw.com/chl/208e8dcf5c64cbc8bdfb.html)》为根据，以《[中华人民共和国妇女权益保障法](https://www.pkulaw.com/chl/dff2eefbddf155b3bdfb.html)》为主体，包括《[中华人民共和国婚姻法](https://www.pkulaw.com/chl/ff6577b27d4ed3efbdfb.html)》、《[中华人民共和国继承法](https://www.pkulaw.com/chl/37ee1b1a0b11733cbdfb.html)》、《[中华人民共和国劳动法](https://www.pkulaw.com/chl/15fe2d94afee49a2bdfb.html)》、《[中华人民共和国母婴保健法](https://www.pkulaw.com/chl/ea633acd04298a40bdfb.html)》、《[女职工劳动保护规定](https://www.pkulaw.com/chl/133408887a349cdabdfb.html)》等法律、行政法规和地方性法规在内的一整套保障妇女权益和促进妇女发展的法律体系；建立健全了与之相应的保障妇女权益的组织机构；采取了有力的措施，有效地推动了妇女事业的发展。改革开放１７年来，我国妇女受教育水平不断提高；就业人数大幅度上升；生活状况日益改善；在社会和家庭中的地位以及参与国家和社会事务管理的程度不断提高。广大妇女积极投身改革开放和现代化建设，为经济发展和社会进步做出了巨大的贡献。所有这些，充分体现了社会主义制度的优越性，为我国妇女的进一步发展奠定了坚实的基础。
　　但是，由于我国是发展中国家，受经济和社会发展水平的制约及旧观念的影响，妇女受教育的程度和参与社会发展的程度还不够高；法律上关于男女平等的规定还没有完全落实；社会上歧视妇女的现象仍然存在；贫困地区妇女的生活状况有待改善。我国妇女发展的任务还很艰巨。
　　当今世界正处在历史性的大变动之中；国际竞争日趋激烈。世界范围的经济、文化和科学技术的竞争，归根到底是民族素质的竞争。妇女的素质影响到民族的素质，妇女的发展水平影响着一个国家的综合国力。在２１世纪即将到来之际，妇女问题更是举世关注的焦点之一，以行动谋求平等、发展与和平，已成为整个国际社会不可逆转的潮流。我国政府已经对有关妇女权利和妇女发展的国际公约作出的庄严承诺。

　　三、在邓小平同志建设有中国特色社会主义理论指导下，我们党和国家确定了基本实现现代化分“三步走”的战略步骤，并明确要在本世纪末实现第二步战略目标。今后几年，既是我国推进改革开放和现代化建设的重要时期，也是我国妇女进步与发展的重要时期。在今后几年中，妇女发展的任务是：动员和组织全国各族妇女投身改革开放和社会主义现代化建设，全面提高妇女素质，依法维护妇女权益，进一步提高妇女地位。广大妇女要发扬自尊、自信、自立、自强的精神，在推动社会发展的过程中求得自身的进步与发展。各级政府、各有关部门、各社会团体和各企（事）业单位要充分认识促进妇女参与发展的重要意义，密切配合，采取有力措施，确保实现本纲要的各项目标。

　　主要目标

　　四、到本世纪末，我国妇女发展的总目标是：妇女的整体素质有明显提高，在全面参与经济建设和社会发展，参与国家和社会事务管理的过程中，使法律赋予妇女在政治、经济、文化、社会及家庭生活中的平等权利进一步得到落实。妇女发展的具体目标是：
　　（一）提高妇女参与国家和社会事务决策及管理的程度。
　　－－积极实现各级政府领导班子成员中都有女性，政府部门负责人中女性比例有较大提高。
　　－－女职工比较集中的行业、部门以及企事业单位的领导班子成员中，应多选配一些女性。
　　（二）组织妇女积极参与改革开放和现代化建设，推动社会生产力发展。
　　－－在建立社会主义市场经济体制和调整城乡产业结构，大力发展第三产业的过程中，增加妇女就业人数，扩大妇女就业领域。
　　－－在录用职工时，除国家规定的不适合妇女的工种或岗位外，不得以性别为由拒绝录用妇女或者提高对妇女的录用标准，以保障妇女与男子平等的就业权利。
　　（三）切实保障妇女的劳动权益。
　　－－所有企业（包括外商投资企业、私营企业、乡镇企业），都要认真贯彻实施《[中华人民共和国劳动法](https://www.pkulaw.com/chl/15fe2d94afee49a2bdfb.html)》及有关女职工劳动保护的规定，保护妇女在工作和劳动时的安全和健康，努力改善女职工的劳动条件。
　　－－在全国城乡实现男女同工同酬。
　　－－在全国城市基本实现女职工生育费用的社会统筹。
　　（四）大力发展妇女教育，提高妇女的科学文化水平。
　　－－逐步提高女性接受各级、各类教育的比例，全面提高妇女劳动者的素质，积极培养各类女性专业技术人才。
　　－－全国基本普及九年义务教育，降低适龄女童的失学率和辍学率，使适龄女童的失学率、辍学率均控制在２％以下。
　　－－每年扫除３００万妇女文盲，力争到本世纪末，全国基本扫除青壮年妇女文盲。
　　－－大力发展各级、各类职业教育、职业培训和实用技术培训，提高妇女就业能力。
　　（五）进一步提高妇女的健康水平，保障妇女享有计划生育的权利。
　　－－提高妇幼卫生机构的服务能力及服务质量。
　　－－努力使城乡妇女人人享有卫生保健，包括享受良好的生殖保健服务。
　　－－全国孕产妇保健覆盖率和孕产妇接受健康教育率达到８５％。
　　－－农村新法接生率达到９５％。
　　－－提高农村孕产妇住院分娩率，使孕产妇死亡率在１９９０年的基础上降低５０％。
　　－－育龄期妇女及孕妇的破伤风类毒素的免疫接种率在高发地区达到８５％，消除新生儿破伤风。
　　（六）提倡建立平等、文明、和睦、稳定的家庭。
　　－－发扬中华民族的优良传统，树立社会主义的道德风尚，在家庭内部、邻里之间建立和发展平等、团结、友爱、互助的关系。
　　－－提倡夫妻共同承担家务劳动和抚育子女。利用多种形式，向父母传播正确教育子女的知识与经验。
　　－－反对重婚纳妾。
　　－－坚决制止干涉婚姻自由的行为。
　　（七）有效遏制对妇女的暴力侵害及拐骗、买卖妇女的犯罪行为和卖淫嫖娼违法活动。
　　（八）重视和扶持边远、贫困和少数民族地区妇女发展。到本世纪末，基本解决贫困妇女的温饱问题。
　　－－对贫困地区的１０００万妇女进行文化和生产技术培训，使她们掌握１门以上实用技术。
　　－－平均达到一村一个女农（牧）业技术员。
　　－－扶持发展脱贫示范户２０万个。
　　－－发展以妇女为主的扶贫经济实体２万个，安排贫困妇女就业８０万人。
　　（九）改善妇女发展的社会环境，提高她们的生活质量。
　　－－在全社会倡导文明进步的妇女观，树立尊重妇女、保护妇女的社会风气；教育妇女发扬自尊、自信、自立、自强精神。
　　－－搞好社区服务，发展托幼事业和家务劳动服务事业。
　　－－保护未成年女性、老年妇女及残疾妇女的特殊利益。禁止用人单位招用未满１６周岁的女性。办好各类社会福利院、老人公寓和敬老院。对贫困残疾妇女开展康复扶贫。
　　（十）扩大我国妇女同各国妇女的友好交往，促进世界和平。
　　（十一）建立妇女状况的动态研究、数据采集和资料传播机制。
　　－－建立国家级的妇女数据库。
　　－－在国家统计系统中设立妇女分类统计指标。

　　政策和措施

　　五、政治权利和参与决策
　　－－通过各种途径，提高对妇女政治权利的认识，使妇女平等参与管理国家事务、社会事务，参与重大问题的决策。
　　－－各级政府在制定政策和规划时，要注意听取各级妇女组织的建议和要求，充分考虑妇女的特殊利益，保障她们的合法权益。
　　－－切实做好培养、选拔女干部工作，要制订计划，定期检查，逐级落实。
　　－－加强对女干部的教育和培训，提高她们的参政能力和领导水平。

　　六、就业和劳动保护
　　－－积极开发适合妇女特点的就业领域和就业方式，为妇女提供更多的就业机会。
　　－－发展职业介绍、就业咨询等服务事业，指导妇女就业。
　　－－积极发展农村多种经营，进一步发展乡镇企业，更多地吸收农村妇女就业，有计划地组织贫困地区妇女劳务输出。
　　－－改善企业，特别是私营企业、乡镇企业以及外国投资企业中女职工的劳动条件，加强劳动保护措施。禁止安排女职工从事女性禁忌的劳动；建立健全女职工劳动保护设施；建立企业女职工定期检查身体和妇科病的制度；加强女职工劳动保护用品的科研工作，不断提高劳动保护水平。
　　－－积极开展劳动监察，依法查处侵犯妇女劳动合法权益的行为。坚决制止企业解除孕期、产期、哺乳期女工的劳动合同、强迫女职工从事超强度劳动、违反男女同工同酬原则，保证女职工在不危害身心健康和生命安全的生产环境中工作。对招用未满１６周岁女童工的单位或个人，必须依法制裁。
　　－－把女职工的劳动保护纳入安全生产领导责任制中，作为考核企业负责人业绩的重要内容之一。加强对女职工进行劳动保护法规、政策的宣传教育，提高女职工的自我保护意识。
　　－－改革女职工生育保障制度。将女职工生育保险费用由企业管理逐步改为社会统筹管理，这项改革由国有企业逐步扩展到所有企业。

　　七、教育与职业培训
　　－－各级政府要认真贯彻执行国务院《[扫除文盲工作条例](https://www.pkulaw.com/chl/87a77c05932fcfa2bdfb.html)》，把扫除边远、贫困和少数民族地区的青壮年妇女文盲作为重点，从各地经济、文化发展不平衡的实际出发，因地制宜，分类指导。
　　－－各级政府要认真贯彻执行《[中华人民共和国义务教育法](https://www.pkulaw.com/chl/27bc0f58a13f5a17bdfb.html)》，将普及义务教育与发展生产力、克服旧习俗和旧观念结合起来，创造有利于女童受教育的社会环境。对边远、贫困和少数民族地区，在政策和资金等方面要给予支持和扶持。采取办女童班等多种办学形式，为贫困地区女童的入学提供便利条件。各级政府应积极帮助解决女童因家庭经济困难不能入学的实际问题。
　　－－逐步提高女性接受中等专业技术教育和高等教育的比例。各类中等专业学校和高等院校在招生工作中，除国家规定的特殊专业外，必须坚持男女平等录取的原则。
　　－－充分利用各类成人学校、职业学校的办学条件，根据社会发展的需要和妇女的特点，在城乡妇女中大力开展不同层次、不同形式的职业教育和职业培训。

　　八、卫生保健
　　－－建立健全各级妇幼卫生机构。加强乡卫生院的产科建设，改善条件及设施，使其具备接生及急救能力。努力提高农村孕产妇住院分娩率，提高农村家庭接生中的新法接生率。到２０００年，使乡级妇幼卫生人员产科急救知识及产科技能培训覆盖率达到８５％，贫困地区村级接生员复训率达到８０％。
　　－－建立妇幼卫生监测网络和常规报告系统。建立和健全孕产妇死亡、婴儿死亡、肉眼可见残疾儿的报告制度。
　　－－开展孕产妇系统保健。预防孕期、产期及产褥期母体和胎儿、围产儿常见疾病的发生。普及新生儿复苏技术，降低早期新生儿死亡率。
　　－－提高妇女健康教育覆盖率。针对妇女一生中不同时期的生理和心理特点，对处于女童期、青春期、生殖调节期、围绝经期、老年期的妇女分别进行健康教育，传播性科学知识、自我保健知识与育儿知识，促进妇女身心健康，发挥妇女在家庭保健方面的作用。
　　－－开展妇女病筛查和防治工作，重点筛查和治疗严重危害农村妇女健康的疾病。
　　－－开展新生儿破伤风高危地区孕产妇及育龄妇女的破伤风类毒素接种工作。在广大妇女中普及儿童计划免疫知识，进一步降低相应疾病发生率和死亡率。
　　－－采取食盐加碘、服用碘油丸等方法，保证妇女体内对碘元素的需要。到２０００年，需要补用碘油的新婚妇女、孕妇９５％能补用碘油，基本消除妇女因孕期及哺乳期缺碘所导致的儿童智力损害。
　　－－－－改善生态环境，采取治水、改水、改灶等方法，控制高氟地区对妇女健康带来的危害。加强对氟斑牙、氟骨症病人的治疗。

　　九、计划生育
　　－－广泛、深入、持久地开展全民性的人口与计划生育的宣传教育。在广大妇女中宣传我国的基本国情和基本国策，引导她们转变婚育观念，树立晚婚晚育，少生优生，生男生女都一样的新观念。
　　－－通过各种途径向广大妇女普及避孕节育、优生优育、妇幼保健方面的科学知识。积极推动遗传病咨询、母婴保健、新生儿筛查技术工作。到２０００年，使先天性病残儿发生率在１９９０年的基础上减少１／２。
　　－－提高计划生育技术。积极研究开发新的安全、有效、方便的避孕药具和节育技术。改进和完善现有的避孕节育技术和方法，降低副作用，减少并发症，提高可接受性，保护妇女的生殖健康。到２０００年，节育手术并发症发生率控制在１０／１００００以下。
　　建立与健全方便群众的基层计划生育技术服务和药具供应网络，为育龄夫妇提供各种可供自由选择的安全、有效、方便的避孕药具和节育技术服务，积极开展对避孕节育的指导，提高避孕节育普及率及有效率。

　　十、法律保护
　　－－制定与《[中华人民共和国宪法](https://www.pkulaw.com/chl/208e8dcf5c64cbc8bdfb.html)》、《[中华人民共和国妇女权益保障法](https://www.pkulaw.com/chl/dff2eefbddf155b3bdfb.html)》相配配套的、切合实际、具有可操作性的行政法规，或制定相应的政策措施，使维护妇女权益的法律更切实可行。
　　－－提高司法和行政执法队伍的素质，充实力量，加强领导，严格监督，确保维护妇女权益的法律、法规得到全面实施。对不严格依法办事，知法犯法，损害妇女权益的行为要依法追究。
　　－－在全国范围内，特别是在广大农村和偏远、贫困地区，继续深入持久地宣传和贯彻执行《[中华人民共和国婚姻法](https://www.pkulaw.com/chl/ff6577b27d4ed3efbdfb.html)》及有尖法律、法规。加强婚姻登记管理工作，采取有效措施，制止早婚、买卖婚姻、近亲结婚等违法婚姻。
　　－－保护妇女的人身权利和一切合法财产权利，及时查处侵害妇女权益的民事案件。
　　－－严厉打击拐骗、买卖、遗弃、虐待、迫害、污辱妇女等犯罪活动，维护妇女的人身安全和人格尊严。坚决取缔卖淫嫖娼活动，扫除社会丑恶现象。
　　－－严肃查处溺弃、买卖、残害女婴的犯罪活动。严禁利用现代医学技术进行非医学原因的胎儿性别鉴定。打击破坏计划生育的违法犯罪行为。
　　－－利用多种渠道和形式，广泛深入地进行法制宣传教育，增强全民法制观念；尤其要引导、帮助广大妇女树立牢固的法律意识，自觉利用法律武器维护自身的合法权益。
　　－－保护妇女合法的控告、申诉权。健全妇女信访的接待与处理制度，防止出现互相推诿，久拖不决的现象。开展法律咨询及代理服务，为妇女群众排忧解难，为受害妇女伸张正义。

　　十一、改善妇女发展的社会环境
　　－－向全社会宣传妇女在创造人类文明、推动社会发展中所发挥的伟大作用；宣传妇女与男子具有同等的人格和尊严、同等的权利和地位；宣传有自尊、自信、自立、自强精神的女性；制止影视、书报刊中对妇女形象的贬低和污辱性描绘。改变社会对女性的歧视和偏见，增进全体公民对妇女合法权益的认识。
　　－－依法保护妇女在家庭中的平等地位，坚决制止家庭暴力。加强家庭文化建设，提高家庭成员的素质。继续开展将思想道德教育、学习科学技术、活跃家庭文化生活、促进家庭经济发展融为一体的家庭文化建设活动，倡导文明、科学、进步的生活方式，促进家风、民风、社会风气的好转。
　　－－加强妇女培训和活动场所的建设。积极组织妇女参加各种文化、科学知识的学习和健康的文体活动。
　　－－继续加强社区服务，推进托幼事业和家务劳动服务事业，进一步减轻职业妇女的家务负担。
　　－－开展为妇女服务的心理咨询活动，提高妇女的心理素质。
　　－－加强有利于妇女身心健康的专用品及保健品的开发、研制和推广工作。
　　－－扶持各省的社会福利事业，做好城乡无法定扶养义务人、无劳动力、无生活来源的孤残女童、孤老妇女的供养工作。要新建、扩建和改造收养上述妇女的各类社会福利院、老年公寓和敬老院，配备必要的生活、医疗、康复设施。
　　－－大力向妇女宣传保护环境的基本国策，政府支持和鼓励妇女兴办“生态农业工程”、“三八绿色工程”、“水土保持工程”等生态建设活动并给予积极扶持。

　　十二、扶持贫困地区妇女事业的发展
　　－－开展实用技术培训，提高妇女脱贫致富的能力。
　　－－开发适合妇女特点的扶贫项目。
　　－－要特别关注残疾妇女的生活状况，在全社会树立理解、尊重、关心、帮助残疾人的良好道德风尚。妥善安排残疾妇女的生活、康复、教育和劳动就业。

　　组织与实施

　　十三、本《纲要）由国务院妇女儿童工作委员会负责组织实施。国务院各有关部门和各社会团体，要根据《纲要》的要求，结合各自的职责范围，制定具体实施方案，负责实施。

　　十四、实施《纲要》是各级政府义不容辞的重要职责。各省（自治区、直辖市）人民政府要在《纲要》的指导下，结合实际情况制订本地区的妇女发展规划，并将其纳入当地经济和社会发展的总体规划，统一部署，统筹安排。要建立目标管理责任制，把《纲要》的落实情况作为各省（自治区、直辖市）、地（市）、县、乡政府主要负责人和主管负责人政绩考核的内容之一。

　　十五、各级政府都要逐步增加用于妇女事业的资金。加强和完美社会化服务体系建设，从物质、信息、技术、贷款等方面更好地支持妇女参与经济活动。鼓励社会各界赞助妇女事业。

　　监测与评估

　　十六、建立健全劳动监察、卫生监测、教育督导、统计评估、法律监督机构，完善监测机制，以确保《纲要》总目标的实施。

　　十七、要加强国家级的妇女发展综合统计，建立妇女数据库，增设性别统计指标，做好有关妇女的信息采集、整理、反馈和交流工作，为预测发展趋势、制订规划、科学决策、检查评估等提供依据。

　　十八、要建立国家级的妇女状况监测系统，制定切实可行、科学规范的监测评估方案，全面地、动态地监测妇女发展状况。

　　十九、为了解和评估《纲要》的实施情况，要建立定期检查、审评制度，以便及时发现问题，采取相应的对策。在实施《纲要》的过程中，要采用多种调查方法，全面、系统、及时地反映妇女状况的发展和变化，进行有重点的专题评审和中期评审。到２０世纪末，要进行终期全面评审，做好实施《纲要》的总结和评估，并制定２１世纪的妇女发展纲要。

© 北大法宝：（[www.pkulaw.com](https://www.pkulaw.com/)) 专业提供法律信息、法学知识和法律软件领域各类解决方案。北大法宝为您提供丰富的参考资料，正式引用法规条文时请与标准文本核对。
欢迎查看所有[产品和服务](https://www.pkulaw.com/helps/2.html)。法宝快讯：[如何快速找到您需要的检索结果？](https://www.pkulaw.com/helps/70.html)[法宝](https://www.pkulaw.com/helps/69.html)V6有何新特色？

扫描二维码阅读原文

**原文链接：**<https://www.pkulaw.com/chl/33670a50094f0c40bdfb.html>
